# *In vivo* photoacoustic imaging of swine ureters injected with methylene blue

**DOI:** 10.1117/1.JBO.31.3.036004

**Published:** 2026-03-14

**Authors:** Junior Arroyo, Junhao Zhang, Jiaxin Zhang, Nethra Venkatayogi, Manik Kakkar, Amanda Maxwell, Kathleen Gabrielson, Muyinatu A. Lediju Bell

**Affiliations:** a Johns Hopkins University, Department of Biomedical Engineering, Baltimore, Maryland, United States; b Johns Hopkins University, Department of Electrical and Computer Engineering, Baltimore, Maryland, United States; c Johns Hopkins University, Department of Computer Science, Baltimore, Maryland, United States; d Johns Hopkins University, Department of Molecular and Comparative Pathobiology, Baltimore, Maryland, United States

**Keywords:** photoacoustic imaging, ureter, methylene blue, gynecology, urology, hysterectomy, renal biomarkers

## Abstract

**Significance:**

Ureteral injuries represent a major concern during a range of surgical procedures, due to the proximity of the ureter to target surgical structures. Intraoperative identification of the ureter is critical to prevent this accidental damage.

**Aim:**

We demonstrate the first known *in vivo* photoacoustic imaging of the ureter in swine following intravenous administration of FDA-approved methylene blue, enabled by a software-hardware integration that has not been previously reported in the literature.

**Approach:**

Photoacoustic channel data from the ureters of two swine were acquired using a Vevo F2 ultrasound system and an Opotek Phocus Mobile laser. Images were beamformed using a delay-and-sum algorithm. Photoacoustic image quality was evaluated using contrast, signal-to-noise ratio (SNR), and generalized contrast-to-noise ratio (gCNR) metrics, measured 10 to 80 min after methylene blue injection.

**Results:**

Across the 10- to 80-min imaging window, median contrast (3.46–11.43 dB), SNR (2.84–6.99), and gCNR (0.27–0.64) confirmed sustained ureter visibility with methylene blue. Maximum image quality was observed 20- to 30-min after methylene blue injection, with significantly higher contrast, SNR, and gCNR values compared with earlier or later time points (p<0.05).

**Conclusions:**

*In vivo* results demonstrate that methylene-blue-enhanced photoacoustic imaging can visualize the ureter over a time duration that is consistent with the length of surgical procedures, providing initial feasibility for real-time photoacoustic-guided surgery applications.

## 1 Introduction

Accidental ureteral injury is a concern during a range of surgical procedures (e.g., hysterectomy,^1^ cesarean delivery,^2^^,^^3^ myomectomy,^4^ oophorectomy,^5^ presacral neurectomy,^6^ prostatectomy,^7^ urologic surgeries^8^). The risk of injury is heightened in complex cases wherein the ureter resides in close proximity to surgical structures, typically induced by clamping, clipping, or cauterizing surrounding or overlapping structures,^9^ or dissecting adjacent structures.^10^^,^^11^ Across surgical subspecialties, hysterectomy accounts for 54% of iatrogenic ureteral injuries,^12^ with gynecologic procedures overall responsible for 64% to 82% of cases,^13^ while urologic surgery accounts for ∼11% to 30% of injuries,^13^ and radical prostatectomy accounts for <1.6% of injuries.^14^ Critically, 50% to 70% of ureteral injuries go undetected during surgery,^15^ often resulting in serious postoperative complications and morbidity, such as renal insufficiency, loss of renal function, the need for additional interventions, prolonged hospitalizations, and in severe cases, patient death.^16^^,^^17^

Photoacoustic imaging is a nonionizing hybrid imaging modality that leverages the photoacoustic effect to visualize tissues based on their optical properties. When nanosecond laser pulses illuminate biological tissue, endogenous chromophores (e.g., hemoglobin) or exogenous agents (e.g., injected dyes) absorb the light, leading to localized heating and rapid thermal expansion. This process generates acoustic pressure waves that are detected by ultrasound sensors. The received acoustic signals are beamformed to generate images that map spatial variations in optical absorption. These variations reveal anatomical structures and functional processes, such as vascular networks, perfusion dynamics, and regions enhanced by exogenous contrast agents.^18^^–^^24^

Building on these advantages, effectiveness with tracing obscured vasculature directly translates to the potential to distinguish vasculature from adjacent anatomical structures. This capability has been explored in the context of photoacoustic-guided hysterectomy, including teleoperated procedures demonstrated with *ex vivo* tissue^25^^,^^26^ and laparoscopic or open hysterectomy procedures performed on human cadavers.^27^ Real-time auditory feedback was additionally incorporated to enhance surgical guidance and reduce the risk of ureteral injury.^16^^,^^26^^–^^30^ In the associated cadaver experiments,^27^^,^^30^ ureter visualization was successfully achieved with the administration of FDA-approved methylene blue as a contrast agent, due to the low optical absorption of urine. Although other studies have demonstrated photoacoustic imaging of organs within the urinary tract (e.g., *in vivo* rat bladders,^31^^,^^32^ cadaver porcine bladders,^33^ *in vivo* human kidneys^34^), to our knowledge, no prior work implements real-time *in vivo* photoacoustic imaging of the swine ureter.

To advance the clinical translation of photoacoustic-guided hysterectomy, it is necessary to demonstrate consistent and reliable *in vivo* visualization of the ureter. While methylene blue is known to provide photoacoustic contrast,^27^^,^^35^ ∼75% of methylene blue is rapidly reduced to its nonfluorescent leucoform ^36^^,^^37^ after intravenous administration. Although the remaining fraction may circulate through the urinary tract, its persistence over time remains uncertain, particularly with prolonged laser exposure. These factors highlight the need to evaluate not only the *in vivo* visibility of the ureter, but its consistent visibility over extended periods of time after methylene blue injection.

The study herein evaluates the feasibility of *in vivo* photoacoustic imaging of the ureter in swine following intravenous administration of FDA-approved methylene blue as a contrast agent with three primary contributions. First, we conducted a non-survival imaging study using intravenous methylene blue injection to provide the first known *in vivo* photoacoustic images of the ureter, which were monitored over an 80 min duration. Second, we quantified ureter visibility using standard image quality metrics. Third, we monitored renal function for a 2-week period prior to imaging, after a survival surgery wherein ureters containing methylene blue were in contact with 750-nm laser light for a 10-min duration. The remainder of this article is organized as follows. Section 2 details the experimental setup and imaging approach. Section 3 presents the photoacoustic images of the ureter and associated image quality metrics. Section 4 discusses our findings within the context of photoacoustic-guided surgery applications. Section 5 summarizes our major contributions.

## 2 Methods

### 2.1 Surgical Procedures and Renal Function Assessment

**Fig. 1:**
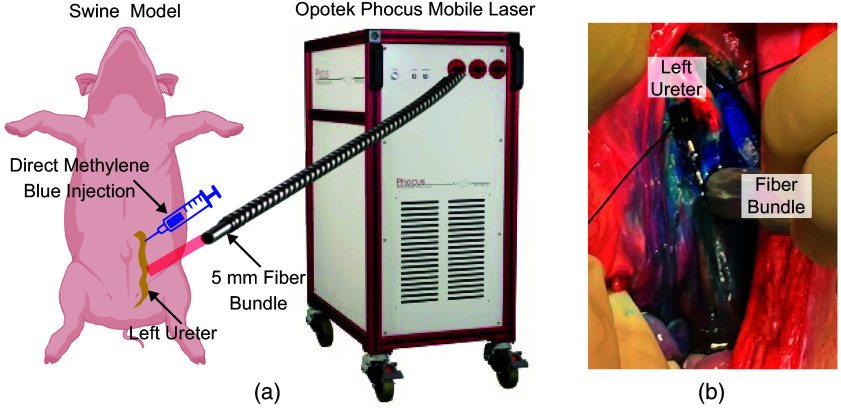
(a) Experimental setup illustrating the use of the Opotek Phocus Mobile laser to expose the swine left ureter. (b) Laser exposure on the left ureter after direct methylene blue injection (2.5 mL, 1755  μM concentration).

The Institutional Care and Use Committee of Johns Hopkins University, an AAALAC-accredited institution, approved the protocol to conduct the *in vivo* studies described herein. Animals and procedures were in compliance with the US Public Health Service’s Policy on Humane Care and Use of Laboratory Animals,^38^ the US Department of Agriculture’s Animal Welfare Act,^39^ and the National Research Council’s Guide for the Care and Use of Laboratory Animals.^40^ We performed a survival surgery to monitor renal function after the delivery of laser energy to ureters containing methylene blue, followed by a non-survival imaging study 2 weeks later in a total of two female Yorkshire swine (weighing 39.9 and 28.6 kg). During the survival procedure, a standard midline laparotomy was performed on the abdomen of each swine, followed by a unilateral rectus abdominis muscle (transrectus) incision to expose the left ureter, as illustrated in Fig. 1(a). Two doses of medical grade methylene blue (Zydus Pharmaceuticals, Pennington, New Jersey, USA) were injected into the left ureter: 0.5 and 2.5 mL, resulting in intraluminal concentrations of 351 and 1755  μM with an assumed ureter lumen volume of $2.22\times 10^{-5}\;m^{3}=22.2\;\mathrm{mL}$^41^ (i.e., ∼50  μM below and ∼4× above the FDA-approved limit,^42^ respectively, when considering the assumptions noted by Wiacek et al.^27^). Following methylene blue injection, the ureters were exposed to laser light with 750-nm wavelength, 5-ns pulse duration, 10-Hz pulse repetition frequency, and 16-mJ mean pulse-to-pulse energy per swine, for a duration of 10 min. Consistent energy was ensured using our previously described energy calibration and monitoring methods.^43^ The energy was delivered using a gas-sterilized 5-mm-diameter optical fiber, as shown in Fig. 1(b).

**Fig. 2:**
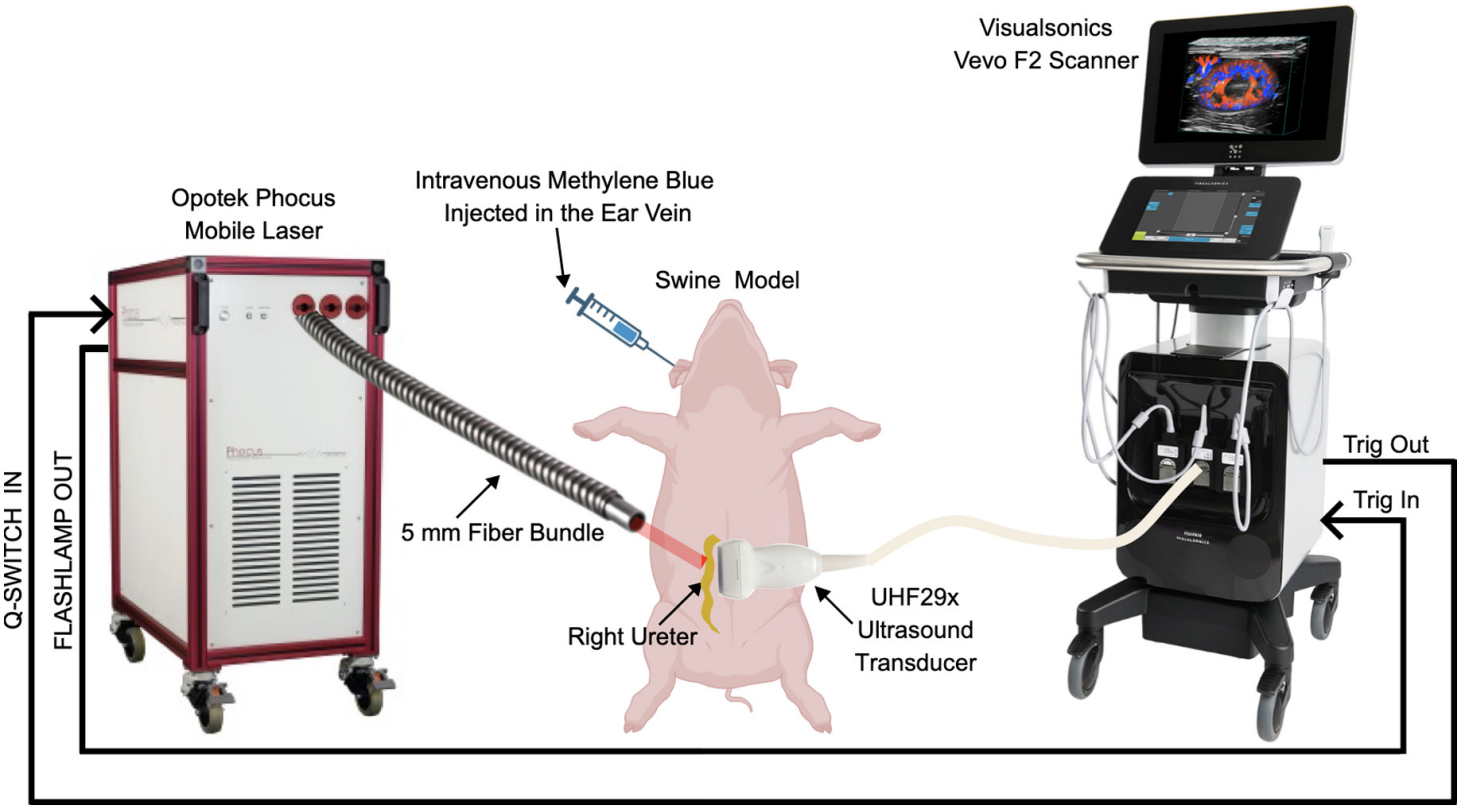
Experimental setup integrating the Opotek Phocus Mobile laser and VisualSonics Vevo F2 ultrasound scanner for *in vivo* photoacoustic imaging of the swine ureter.

Two weeks after the initial surgical procedure, a new standard midline laparotomy was performed on the abdomen of the same two swine, followed by a unilateral rectus abdominis muscle (transrectus) incision to expose the right ureter for the imaging study, as shown in Fig. 2. Methylene blue was administered intravenously through an ear vein using two 10-mL vials (50 mg each), resulting in an intraluminal concentration of ∼400  μM, in accordance with the FDA-approved dosage limit.^27^^,^^41^^,^^42^ Photoacoustic imaging started ∼5  min after intravenous methylene blue administration, to allow for systemic circulation. Blood samples were collected at 1-week intervals to assess renal function using two biomarkers: blood urea nitrogen (BUN) and serum creatinine (SC), which are reliable indicators of kidney function, with elevated levels suggesting potential renal dysfunction.^44^^,^^45^

### 2.2 Integrated Photoacoustic Imaging System

A Phocus Mobile laser (Opotek, Inc., Carlsbad, California, USA) was integrated with a Vevo F2 ultrasound system (FUJIFILM VisualSonics, Toronto, Canada), connected to a 256-element UHF29× linear array ultrasound transducer (15 to 29 MHz bandwidth, 21.33 MHz center frequency) to perform photoacoustic imaging. This integrated Vevo-Opotek system synchronized laser emissions with the ultrasound electronics, allowing the coregistered acquisition of ultrasound and photoacoustic data. The required system connections to image the swine ureter are illustrated in Fig. 2.

### 2.3 Imaging Procedure

**Fig. 3:**
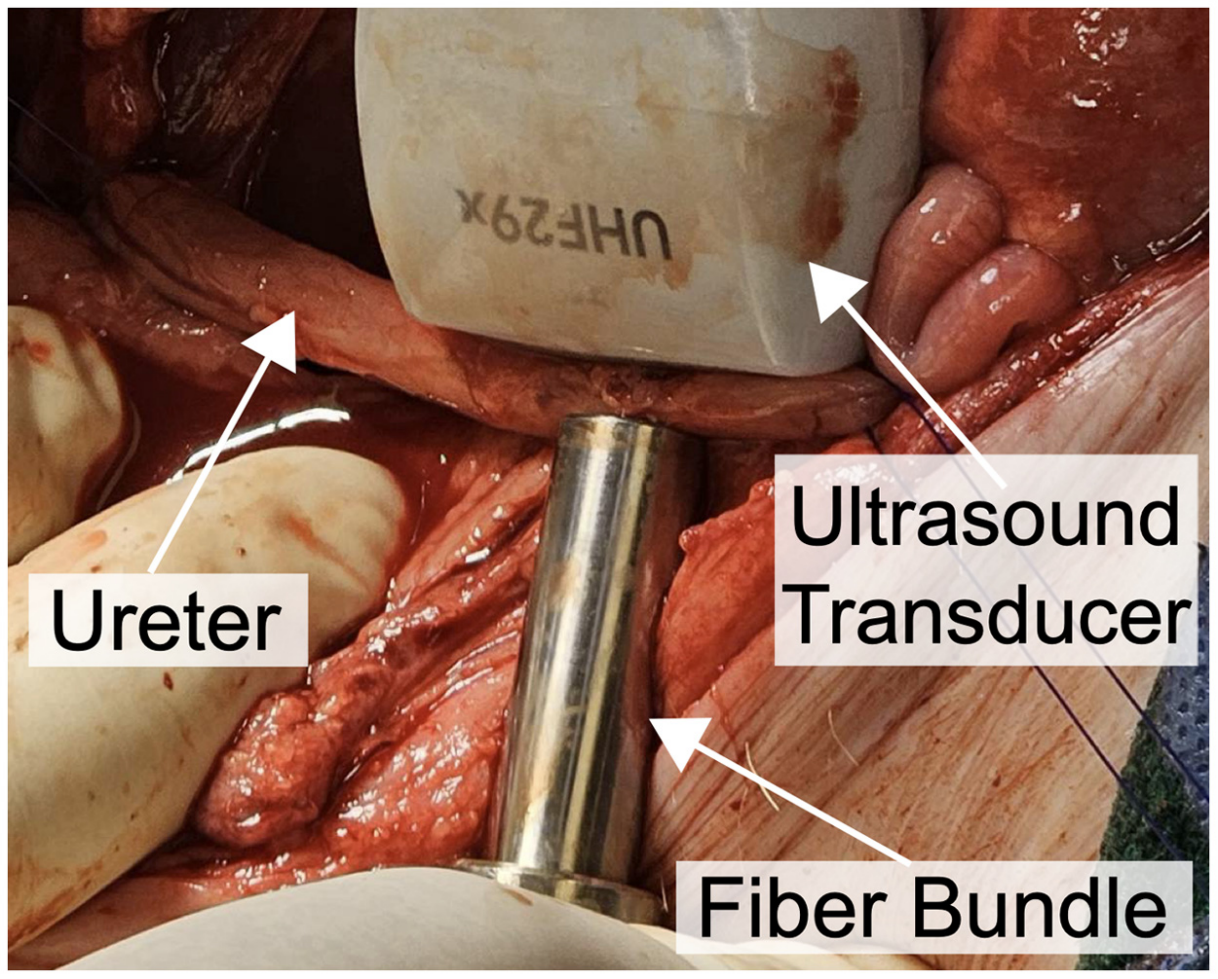
Photoacoustic imaging of the right ureter after systemic methylene blue injection (20 mL, 400  μM concentration). The ultrasound transducer was positioned along the cranial/caudal axis of the ureter to acquire photoacoustic signals.

*In vivo* photoacoustic imaging of the right ureter was performed in two swine, with a photograph of the setup shown in Fig. 3. Laser light was delivered with a 5-mm-diameter optical fiber bundle. The lateral dimension of the ultrasound transducer was oriented along the cranial/caudal axis of the ureter, while the optical fiber was placed in contact with the adventitia (outermost surface of the ureter). Photoacoustic images were acquired after freehand adjustment of the light source position to maximize the photoacoustic signal. The mean pulse-to-pulse laser energy measurements before and after photoacoustic imaging were 15.89 and 12.47 mJ, respectively, for the first swine and 15.61 and 6.78 mJ, respectively, for the second swine.

The imaging protocol was identical in both animals, except for the time window for data acquisition. For the first swine, imaging was limited to 10- to 30-min post methylene blue injection. For the second swine, imaging was performed within 60- to 80-min after methylene blue injection. This combination provided temporal coverage over an extended 10- to 80-min interval, enabling the assessment of *in vivo* ureter visualization at both early and delayed time points after methylene blue injection.

Coregistered raw photoacoustic channel data, beamformed radiofrequency (RF) ultrasound data, postprocessed photoacoustic image data, and postprocessed ultrasound B-mode image data were simultaneously acquired as the light source and ultrasound probe were held in place at 10-, 20-, 30-, 60-, 70-, and 80-min intervals after methylene blue injection. Postprocessed ultrasound and photoacoustic image data from the system were used to create videos, and raw photoacoustic channel data were used to create images for signal analysis, after implementing delay-and-sum (DAS) beamforming using the parameters reported in Table 1. Beamformed photoacoustic and ultrasound RF data were then envelope-detected, normalized to the brightest pixel in each image, and log compressed prior to B-mode image display.

**Table 1:** Delay-and-sum beamforming parameters.

Parameter	Value
Transducer aperture width	23 mm
Number of receive elements	256
Pitch	0.09 mm
Center frequency	21.33 MHz
Sampling frequency	96 MHz
Speed of sound	1538.5 m/s

### 2.4 Image Quality Metrics

A segmentation-based frame selection strategy was employed, considering variability in ureter visualization across acquired data, which was introduced by factors such as physiological motion, acoustic decoupling, and misalignment among the laser illumination location, imaging plane, and ureter. To ensure ureter-specific photoacoustic signal analysis, manual segmentation masks delineating the ureter lumen in ultrasound B-mode images (if visible) and regions containing photoacoustic signal in corresponding coregistered photoacoustic images (if present) were first generated. Frames were retained when segmented photoacoustic signals spatially overlapped the segmented ureter region, indicating effective laser delivery to the ureter within the imaging plane, and frames with stationary signal duplicates were excluded.

Contrast, signal-to-noise ratio (SNR), and generalized contrast-to-noise ratio (gCNR)^46^^–^^48^ of photoacoustic signals were calculated using the following equations:

$$\text{Contrast}=20\;\log_{10}(\frac{\mu_{t}}{\mu_{b}}),$$ (1)

$$\mathrm{SNR}=\frac{\mu_{t}}{\sigma_{b}},$$ (2)

$$\mathrm{gCNR}=1-\overset{N-1}{\underset{k=0}{\sum }}\;\min\{h_{t}(x_{k}),h_{b}(x_{k})\},$$ (3)

where $\mu_{t}$ is the mean, $\sigma_{b}$ is the standard deviation, and $h_{t}$ and $h_{b}$ are the histograms of the photoacoustic signal amplitudes (after envelope detection and normalization) within rectangular (i.e., 3  mm×0.6  mm) regions of interest (ROIs) associated with the target (denoted by subscript t) and background (denoted by subscript b), N=256 is the number of histogram bins, centered at $\left\{x_{0},x_{1},\ldots ,x_{N-1}\right\}$, and k is the index of the bin.

To select photoacoustic target ROIs in each retained frame, the intersection of manually segmented masks from coregistered ultrasound and photoacoustic images (described above to select retained frames) defined a spatially constrained search region in which the brightest photoacoustic pixel was identified. An initial rectangular target ROI (3  mm×0.6  mm) was first centered on the brightest photoacoustic pixel. If this initial ROI extended beyond the segmented ureter region, the ROI location was iteratively updated to the nearest pixel in eight possible directional offsets (i.e., laterally, axially, or diagonally). At each location, the eight directional offsets were evaluated to determine if (1) the ROI boundaries remained within the image dimensions, (2) the brightest pixel remained within the ROI, and (3) ROI pixels intersected the ureter mask, until the updated ROI was entirely contained within the ureter mask.

The first ROI position to satisfy the three conditions noted above was selected to prioritize minimal displacement from the initial ROI position. When multiple candidate positions satisfied the criteria, the fixed selection order was left then right lateral shifts, followed by proximal then distal axial shifts, followed by left proximal, left distal, right proximal, then right distal diagonal shifts. The associated background ROI of identical size was placed at the same image depth as the target ROI, at a fixed lateral distance of 0.54 mm from the right edge of the photoacoustic image. Using this approach, 28, 43, 39, 64, 48, and 65 frames were retained for quantitative analysis at 10-, 20-, 30-, 60-, 70-, and 80-min intervals after methylene blue injection, respectively.

When displaying boxplots to represent image quality results, the horizontal line within and the height of each box represent the median and interquartile range, respectively. The vertical lines above and below each box extend to the maximum and minimum values, excluding outliers, defined as values exceeding 1.5 times the interquartile range, which appear as data points.

### 2.5 Statistical Analyses

To compare the statistical significance of contrast, SNR, and gCNR values measured at 10-, 20-, 30-, 60-, 70-, and 80-min intervals after methylene blue injection, independent samples were first assumed, due to slight variations in imaging location caused by both optical fiber and ultrasound transducer repositioning. Each time point produced 28 to 65 valid datasets (i.e., contrast, SNR, and gCNR measurements, as described in Sec. 2.4), providing sufficient samples for nonparametric statistical comparison. The Shapiro-Wilk test was then applied to determine the normality of each dataset distribution. As the datasets were predominantly non-normally distributed and included unequal sample sizes, group comparison across time points was performed using a rank-based, nonparametric Kruskal–Wallis test. When the Kruskal–Wallis test indicated that at least one group differed in its distribution of ranks, Dunn’s post hoc test was used to identify the pairwise differences. The significance threshold was set at p<0.05. Analyses were performed using MATLAB R2024b software (MathWorks, Natick, Massachusetts, USA).

## 3 Results

### 3.1 Blood Biomarkers Results

**Fig. 4:**
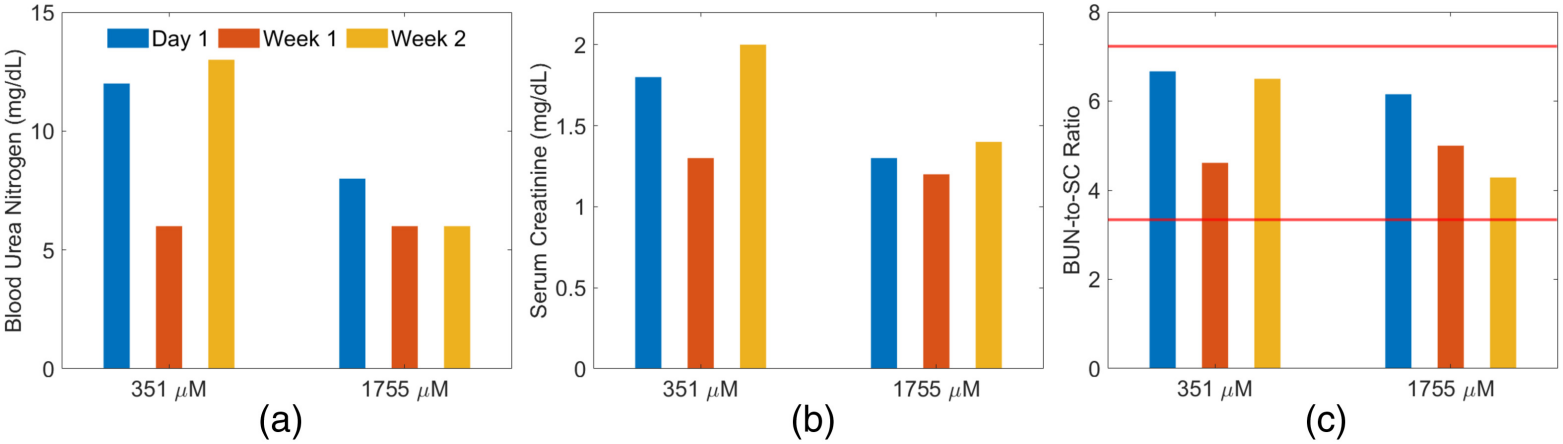
Blood biomarker results from two swine for (a) blood urea nitrogen (BUN), (b) serum creatinine (SC), and (c) BUN-to-SC ratio. The red solid lines represent the upper and lower bounds for the range of normal values for these parameters.

Figure 4 shows the results of the blood biomarkers collected at 1-week intervals. The majority of the BUN [Fig. 4(a)] and SC [Fig. 4(b)] values were within or near physiological limits (BUN: 2 to 13  mg/dL; SC: 0.6 to 1.8  mg/dL),^49^ with the exception of one SC measurement from the first swine, at week 2, which resided outside of this range (i.e., 2.0  mg/dL), likely due to factors such as dietary and physiological conditions.^50^ The BUN-to-SC ratio [Fig. 4(c)] is often considered to be more informative of renal health,^51^ and this ratio resided within the normal range of 3.33 to 7.22.^49^

### 3.2 Photoacoustic Imaging of the Ureter

**Fig. 5:**
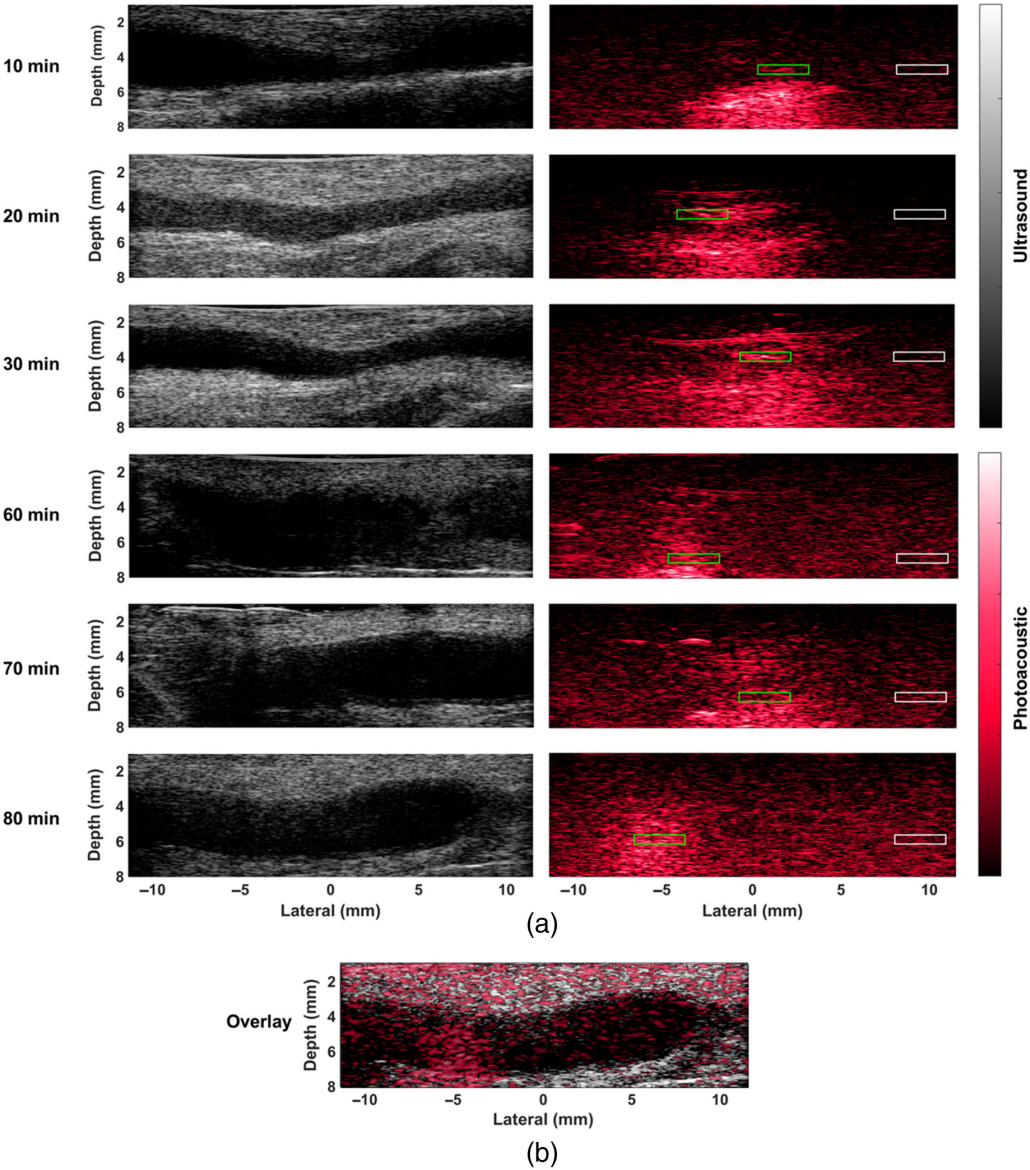
(a) Representative B-mode and photoacoustic images of the swine ureter acquired at time points 10, 20, 30, 60, 70, and 80 min after methylene blue injection. B-mode images are shown with a dynamic range of 45 dB. Photoacoustic images are shown with dynamic ranges of 30, 30, 30, 30, 25, and 25 dB from top to bottom. The target (green rectangle) and background (white rectangle) regions of interest (ROIs) were used to calculate image quality in the photoacoustic images. (b) Photoacoustic image overlaid on coregistered B-mode image corresponding to the data acquisition at the 80 min time interval after methylene blue injection, taken from Video 1. The B-mode image and photoacoustic image are shown with a dynamic range of 15 and 18 dB, respectively (Video 1, MP4, 11.5 MB [URL: https://doi.org/10.1117/1.JBO.31.3.036004.s1]).

Figure 5(a) shows representative B-mode and photoacoustic images of the right ureter acquired with the ultrasound transducer oriented along the cranial/caudal axis of the ureter at six time points (i.e., 10-, 20-, 30-, 60-, 70-, and 80-min after methylene blue injection). Photoacoustic signals were observed in the adventitia lining the ureter and in the ureteral lumen. These representative images have contrast values of 3.55, 12.8, 11.03, 6.13, 3.44, and 7.73 dB, SNR values of 3.09, 8.32, 6.79, 4.02, 2.86, and 4.73, and gCNR values of 0.28, 0.69, 0.60, 0.44, 0.28, and 0.57 at 10-, 20-, 30-, 60-, 70-, and 80-min intervals after methylene blue injection, from top to bottom, respectively. Therefore, the signal of interest is visible within the contrast, SNR, and gCNR ranges of 3.44 to 12.8 dB, 2.86 to 8.32, and 0.28 to 0.69, respectively.

Figure 5(b) overlays a photoacoustic image on its corresponding B-mode image, taken from a representative frame in Video 1. The video displays real-time photoacoustic images overlaid on coregistered B-mode images, demonstrating that the photoacoustic signal is localized within the ureter lumen despite minor movement of the ultrasound transducer or optical fiber bundle. In addition, when the ureter lumen is not visible, no photoacoustic signal was observed. These results confirm that the measured photoacoustic signals are due to methylene blue uptake.

**Fig. 6:**
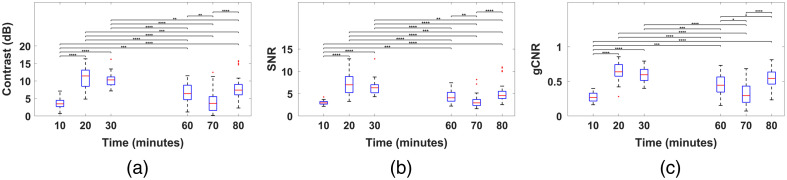
Box-and-whisker plots of the metrics (a) contrast, (b) signal-to-noise ratio (SNR), and (c) generalized contrast-to-noise ratio (gCNR). Statistically significant differences are indicated with *, **, ***, and ****, corresponding to p-values of p<0.05, p<0.01, p<0.001, and p<0.0001, respectively.

Figure 6 shows box-and-whisker plots of the measured contrast, SNR, and gCNR distributions. The maximum values for each metric occur within 20 or 30 min of methylene blue injection, with statistically significant differences relative to earlier or later time points observed in most cases. Over the entire 10- to 80-min time duration, the median contrast (ranging 3.46 to 11.43 dB), SNR (ranging 2.84 to 6.99), and gCNR (ranging 0.27 to 0.64) indicate successful visibility of methylene blue within the ureter.

## 4 Discussion

We successfully produced the first known photoacoustic images of *in vivo* swine ureter using methylene blue as a contrast agent (Fig. 5). There was no renal function impairment from methylene blue administration followed by laser application for 10 uninterrupted minutes, as demonstrated by blood biomarkers results (Fig. 4). In addition, the ability to maintain ureter visibility up to 80-min after methylene blue injection, without redosing, is promising for photoacoustic-guided surgery, supporting the use of methylene blue for prolonged *in vivo* imaging of the ureter (Fig. 6). Although exposed ureters are visible in the photoacoustic images with the presented approach (Fig. 5), as required for these technological validation studies, our technology is ultimately intended to assist in complex cases in which the ureter is hidden by tissue, such as endometriosis, pelvic malignancies, or extensive scar tissue.^27^^,^^30^

Our key findings summarized above align with and complement previous efforts to enhance *ex vivo* ureter visualization of human cadavers, using FDA-approved methylene blue as a contrast agent.^26^^–^^28^ To provide additional context, Gonzalez et al. ^52^ demonstrated gCNR values ranging from 0.49 to 0.89 for cardiac catheter imaging, and Gubbi et al.^53^ reported that *in vivo* gCNR values ≥0.56 were sufficient to ensure reliable segmentation during catheter tracking. These findings are consistent with the gCNR values observed in Fig. 6, offering additional support that our approach can provide acceptable target visibility during real-time surgical applications.

The observed variability in image quality metrics at each time point in Fig. 6 is likely due to multiple factors, such as manual adjustments during ultrasound transducer and optical fiber manipulation, positional drift during free-hand scanning, peristalsis, changes in ureter curvature, and other involuntary tissue motion over time (e.g., due to fluid accumulation in the ureters). Changes in the relative angle, tissue coupling, and distance between the optical fiber and tissue surface could have also altered the local distribution of light, ultimately affecting photoacoustic signal amplitudes. It is also known that various image quality metrics can produce inconsistent results.^47^ Importantly, there was a 3.42 to 8.83 mJ decrease in mean energies before and after each experiment (noted in Sec. 2.3), which could also contribute to the decrease in image quality metrics over time. As these factors are inherent to the imaging process, environment, and technology design, the associated variability is expected to persist when this technology is translated to humans.

The ureter was surgically exposed during these experiments, with the optical fiber placed in direct contact with the tissue. Although this configuration prevented extended depth assessments, photoacoustic imaging can generally achieve penetration depths of several centimeters in soft tissue,^22^^,^^54^ whereas fluorescence and white-light imaging modalities are fundamentally limited by light scattering and absorption, restricting imaging depths to a few millimeters.^55^ Relevant prior work has shown that fluorescence-based ureter visualization using methylene blue in lower abdominal surgeries has 3- to 5-mm-depth penetration, with deeper ureter identification posing challenges.^56^

Limitations of our study include the bulkiness of the imaging devices and the energy levels that exceeded safety limits reported for skin. The form factor of the required optical components can potentially be minimized with strategies such as motion-based light delivery,^57^ integration of fiber-based illumination with surgical tools,^25^^,^^27^^,^^58^ and the use of laparoscopic ultrasound transducers for intraoperative sensing.^27^ The American National Standards Institute (ANSI) reports a maximum allowable pulse energy per unit area of $25.2\;\mathrm{mJ}/{\mathrm{cm}}^{2}$ when imaging through skin with an optical wavelength of 750 nm. Although safety limits specific to ureter tissue have not yet been defined, the maximum permissible exposure (MPE) for skin is commonly used as a reference for internal tissues during surgical and interventional guidance demonstrations.^58^ The fluence values used in our study (i.e., 34.53 to $80.93\;\mathrm{mJ}/{\mathrm{cm}}^{2}$, corresponding to 6.78 to 15.89 mJ energy per pulse, respectively), exceeded the $25.2\;\mathrm{mJ}/{\mathrm{cm}}^{2}$ ANSI safety limits for skin by a factor of 1.4 to 3.2. In a clinical setting, fascia, fat, and other overlying tissues attenuate the incident light, resulting in lower expected fluence values at the ureter surface. A comprehensive assessment that includes direct evaluation of the ureteral wall and surrounding structures will be helpful to assess the degree of restrictiveness of current safety limits, as previously assessed for swine liver^43^^,^^59^ and to determine generalizability to clinical scenarios with the ureter hidden by overlying tissues, adhesions, or complex pathology.

Future work will focus on advancing this technology toward clinical translation. Key steps include assessing the thermal safety of the ureter using the current imaging parameters (i.e., laser energy and exposure time), developing minimally invasive imaging probes compatible with laparoscopic procedures to facilitate practical implementation, and validating imaging performance with overlying tissues to demonstrate robustness under clinically relevant conditions. In addition, optimization of methylene blue dosage will be required to maximize ureter contrast and minimize extraneous target exposure. Collectively, these future directions will be essential to advance the technology from preclinical demonstration to safe and effective clinical implementation.

## 5 Conclusion

We successfully demonstrated the first known *in vivo* photoacoustic imaging of the ureter in a swine model using intravenous methylene blue as a contrast agent. After successfully integrating an Opotek Phocus Mobile laser and with a Vevo F2 ultrasound system, we acquired coregistered ultrasound and photoacoustic images across multiple time points (i.e., 10- to 80-min after methylene blue injection). Maximum image quality was achieved 20- to 30-min after methylene blue injection with consistent ureter visibility maintained up to 80 min without redosing, indicating prolonged contrast agent retention and sustained imaging contrast. There was also no renal function impairment over a 2-week monitoring period after methylene blue administration and laser application. These findings support the clinical potential of photoacoustic-guided surgery to improve intraoperative ureter identification and reduce the risk of injury.

## Supplementary Material

10.1117/1.JBO.31.3.036004.s1

## Data Availability

Code, data, and materials used in this study are proprietary to the PULSE Lab and can be made available upon reasonable request to the PULSE Lab director (MALB). Fulfillment of such requests requires involvement and signed agreements between users and the relevant office(s) at Johns Hopkins University.
